# COVID-19 crisis management of German ICU clinicians in leadership – a metaphor analysis

**DOI:** 10.3389/fpubh.2023.1160094

**Published:** 2023-08-08

**Authors:** Julia Piel, Madlen Hörold, Susanne Brandstetter, Karl-Philipp Drewitz, Ilona Hrudey, Rudolf Schmitt, Christian Apfelbacher

**Affiliations:** ^1^Institute of Social Medicine and Health Systems Research, Medical Faculty, Otto von Guericke University Magdeburg, Magdeburg, Germany; ^2^University Children's Hospital Regensburg (KUNO) at the Hospital St. Hedwig of the Order of St. John, University of Regensburg, Regensburg, Germany; ^3^Faculty of Social Sciences, University of Applied Sciences Zittau-Görlitz, Görlitz, Germany

**Keywords:** healthcare professionals, COVID-19 pandemic, preparedness, hospital, qualitative study, leadership, crises, staff shortage

## Abstract

The COVID-19 pandemic coincided with an already long-standing crisis in health systems around the world characterized by economic pressure and increasing staff shortage. “Crisis” became a global metaphor to convey collective experiences of the COVID-19 threat. Little is known on how crisis metaphors influence thought and speech on crisis management and the challenging staff situation of intensive care unit (ICU) clinicians in leadership positions and how they act. Therefore, we were interested in (1) which metaphorical concepts ICU clinicians in leadership use to express experiences and strategies in dealing with coinciding crises, (2) how these change over time, and (3) how metaphors in speech reveal self-images of crisis management. We conducted a systematic metaphor analysis focusing on data from three participants of a qualitative interview study with twenty-four healthcare professionals in ICUs in Germany. The participants were interviewed at two time points between April 2020 and March 2021. We identified and reconstructed metaphorical concepts of three interviewees (ICU clinicians in leadership) with regard to the pandemic management, and developed a typology based on the dimensions of mood, modus operandi, location, and scope. The typology consists of eight self-images (protagonists) for the crisis management of ICU clinicians in leadership, such as the figure of the soldier (“to unite everyone behind this flag”), the distributor (“sometimes it is a crazy patchwork [*wahnsinniges Gestückel*]”) or the critic (“we are the fool for everything”). They embody different qualities of a leader and refer to intra- and inter-role conflicts within multiple crisis conditions. Metaphor analysis reveals different self-images of ICU leadership clinicians in relation to crisis management. This illustrates that thinking and perceptions of crisis management may strongly differ between and within leaders and may change over the course of crises. Our findings highlight the need both to improve knowledge on challenges associated with leadership in crises and preparedness, and to support clinicians in their leadership by recognizing and addressing differences and changes in leaders’ self-image.

## 1. Introduction

The COVID-19 pandemic coincided with existing long-term and ongoing crises in health systems around the world: economic pressure and increasing staff shortage had already weakened hospitals years before (1, 2). Thus, in the beginning, the COVID-19 crisis manifested itself in the shortage of critical resources in the intensive care units (ICUs) and exacerbated challenges for healthcare professionals (HCPs) (3). The first COVID-19 wave in Germany occurred in weeks 10–20 of 2020 with a total of 175,013 registered cases throughout the country. Cases were more frequent among older adults and severe courses were observed. The second German COVID-19 wave (week 40 of 2020 to week 8 of 2021) peaked at the end of 2020, with 2,158,013 cases reported and considerably more severe courses in all age groups (4).

Generally, “crisis” describes a situation in which major challenges of adaptation, coordination, and possibly modifications and protection need to be solved in a short time (5). Historically, different disciplines understand crisis as constructed in narratives and, since the 20th century, as constitutive for meanings of economic, political, and social reality (6). Consequently, the concept of crisis also applies to the global spreading of SARS-CoV-2.

In contrast to the long-lasting crisis in health systems, in this article, we define the pandemic as a (societal) crisis by addressing its cyclical structure. Regarding the different waves of infection, we do not identify it as a long-term and lasting state, but consider within this phenomenon partially sequential (seasonal) crises (“crises within crisis”) (7). We define a “crisis cycle” experience characterized by repetitive and changing events. This perspective allows us to address the temporality and dynamics of crisis structures as well as the subjective level of experiencing coinciding crises.

In framing the pandemic as a global emergency and “the outbreak of war” (8), politics, media, and the public made references to military scenarios and natural catastrophes to express collectively shared experiences of anxiety about the global virus spreading (9–13).

Especially at the beginning of the pandemic, we saw unimaginable situations in ICUs in some countries. In the media, scenarios of overcrowded hospitals spread around the world: temporary camps with care equipment to treat a mass of critically ill patients in hospitals, isolated dying SARS-CoV-2 infected persons, and health professionals gasping for breath, due to working with limited resources under ongoing pressure (14).

While the beginning of the COVID-19 crisis in hospitals was characterized mostly by limited protective equipment and limited health care capacities for an unusual increase in the number of critically ill patients, a concrete image of the fundamental crisis in health care systems was missing. Aspects of human resources and working conditions in hospitals became more visible under pandemic conditions in the public (15), especially at the beginning of the pandemic when people applauded on balconies to express their appreciation and sympathy towards HCPs (16). As time passed, however, their visibility diminished and precarious structures in healthcare persist even now.

Politics and the media used a variety of metaphorical concepts to express and order the complex experience of the pandemic that also became part of people’s everyday language (12, 17, 18). The pandemic was portrayed as a health, economic, social, and political crisis, to influence individual behavior and to take preventive action against the pandemic, and in later phases to support the global promotion of vaccination (19).

According to a basic understanding of Aristotle, “a metaphor consists in giving the thing a name that belongs to something else; the transference being either from genus to species (…), or on the grounds of analogy (…). One will accordingly describe evening as the ‘old age of the day’ (…) and old age as the ‘evening’ or ‘sunset of life’” (20). Following this, metaphors are figurative replacements on a linguistic level. Aristotle distinguishes between a literally realistic meaning (“age”) and a poetically figurative description (“sunset of life”).

From a cognitive linguistic perspective, Lakoff and Johnson take a broader stance and define the essence of a metaphor in understanding and experiencing a certain subject in terms of another (21). The main function of a metaphor is the understanding of abstract, emotional, or other experiences. Therefore, it is about a pattern of understanding and experiencing that is transferred to another field. Metaphors help us make sense of our experiences, especially if they are emotional and complex. A metaphor can be the best approach to organize aspects of individual experience in a coherent way (21). Different metaphors can structure different aspects of a single experience or phenomenon.

In the context of what has been described as a “collapsing system” in many countries (22, 23), research has highlighted experiences of changing working conditions, psychological burden, and well-being from the perspective of frontline workers caring for critically ill COVID-19 patients (24–27). While the study of the metaphors of the pandemic (9, 10, 18, 28, 29) as well as clinicians in general (30–35) in the pandemic or on policy-makers in leadership (36) is well developed, research on the use of linguistic images and crises management (CM) by clinicians in leadership positions during the pandemic experience is scant (37). However, participants in our previous study on pandemic experiences of HCPs in German ICUs (38) emphasized the need to explore leadership, too.

This study aims to help filling this gap by exploring the metaphorical expressions of CM in ICU clinicians’ experiences in the co-incidence of the COVID-19 pandemic and staff shortage in health systems. By focusing on metaphors, we explore how words/word groups are transferred from their original context (source area) into a different one (target area) (39). Doing so allows us to reconstruct and summarize subjective and group-specific constructions of CM and their comprehensive description. The leading questions of our analysis were (1) which metaphorical concepts ICU clinicians in leadership roles use to express experiences and strategies in dealing with coinciding crises, (2) how these change over time, and (3) how metaphors in speech reveal self-images of CM.

## 2. Materials and methods

At the beginning of the COVID-19 pandemic, we initiated an exploratory interview study focusing on experience and practices of HCPs in German ICUs during the pandemic. We used a grounded theory approach. Between the end of March 2020 and March 2021 we conducted individual interviews with 39 ICU HCPs, namely 19 nurses, 17 clinicians (nine clinicians with leadership position), and three medical students; 24 participants were interviewed twice. The participants were recruited through e-mail, telephone, professional networks, flyers, and personal contacts. For each interview, a protocol was prepared which reported important aspects. The interviews proceeded without interruptions or complications. In addition, the research group prepared for possible complications (e.g., emotional reactions of the interviewees), for example, by providing contact options for counseling services if necessary. A detailed description on the methodology can be found in Hörold et al. (38).

In our study, we described negotiations of social practice and interaction at the beginning of the COVID-19 pandemic in Germany and found a complex field of ambivalences between habits and the new normality of a “daily routine of preparation” (38). We repeatedly observed the challenging situation faced by the ICU leaders and metaphorical speaking of our participants to express experiences with CM. Therefore, we initiated analyzing metaphors in the interviews and performed a systematic metaphor analysis (40, 41) on interviews with ICU clinicians from the original study sample (38). Since we interviewed our study participants twice, we assumed to observe the progression of CM. Based on our empirical findings – the metaphorical concepts and their common characteristics – we developed a typology of protagonists on CM from the perspective of ICU clinicians in leadership.

### 2.1. Three German ICU clinicians

The three interviewees we focused on with metaphor analysis were clinicians in leadership positions working in ICUs, which we briefly portray below. We chose the three interviewees based on common characteristics. First, the clinicians were in a leadership position in hospitals of centralized care, had similar work experience, and were involved in the same way in structures and processes of working in the ICU during the crisis’ phases. Second, the interviews were conducted at comparable time points (baseline interview May 2020 and the follow-up November/December 2020). Third, the three interviewees were of similar age (midlife) and ethnic background (white individuals). We do not include gender identity and use pseudonyms for our participants (cases 1–3).

Case 1 (C1) is an ICU senior clinician. C1 had more than 10 years of clinical work experience and a similar amount of experience in intensive care. C1 worked in a hospital in a COVID-19 hotspot and considered this time to be incredibly exhausting.

A further participant we interviewed was a head clinician defined as case 2 (C2). C2 had more than 10 years of work experience as a physician. Their perspective on working in the hospital changed from baseline to follow-up interview. While C2 was highly motivated in the first interview, C2 expressed being more vulnerable in the second interview.

Case 3 is the third participant in our study. The senior ICU clinician had more than 20 years of work experience at the time of the first interview. In the baseline as well as the follow-up interview, C3 was critical of the working conditions and the political CM. C3 described their own experiences in managing the first phase as a state between disbelief and anger.

### 2.2. Metaphor analysis: background and practice

We understand metaphors of ICU clinicians as cores of narratives. While narratives are used to deal with divergence in everyday experience, metaphors additionally mediate the meaning of them (42). Metaphors are expressions of strategic complexity reduction with the aim of (cognitively) ordering circumstances in crisis cycles, which are embedded in narratives. This ordering activity is a prerequisite to action. Martin and Lueckenhausen (43) described how people may not just use a single metaphor but several different metaphors to communicate their emotions or thoughts. Therefore, linguistic images represent complex cognitive processes in simple patterns and thus offer models of orientation (41).

Schmitt proposes a systematic approach to the analysis of metaphors that involves successive steps (44). Preparing the first step of metaphor analysis, we distinguished between three types of metaphors: (1) occupation-related metaphors (as an expression of professional practice in a healthcare), (2) metaphors of crisis in the healthcare system, and (3) pandemic-related metaphors. Prevailing occupational metaphors typical for the profession were not the focus of our analysis. We concentrated on newly occurring metaphors but were aware that these may have been influenced by prevailing metaphors. In the first analytical step, we defined CM as the target area (40, 41). We examined both sets of interview data (baseline and follow-up), segmented the text into metaphoric elements, and listed all the metaphors from each case. Based on this preliminary work, we selected the metaphors according to the target area. After that, we thematically sorted the metaphors through an inter- and cross-case comparison. The aim was to gather and order similar source areas of metaphors across the different interviews (39–41) and to build metaphorical concepts. Schmitt also notes that metaphors “(…) do not occur isolated in texts, but build metaphorical concepts being reconstructed” (44). We generated several metaphorical concepts from the three cases. Thus, at a certain point, we noticed a saturation of metaphorical concepts. Schmitt describes this analytical step as the “actual interpretative and reconstructive step” (45). We conceptualized a typology of eight CM protagonists (Table 1). The protagonists embody clinicians’ metaphorical speech representing their tasks in leadership during the pandemic cycles. Each protagonist has a specific inventory of CM strategies. All eight types integrate characteristics of the interviewees, which is why single participants and protagonists are not congruent. Rather, the types are composed of common and different characteristics of the single cases.

**Table 1:** Main source areas and protagonists.

Interview quotes	Source area	Protagonists
“we say ‘red button’. You push it and the person is exploding”	Action movie	Adventurer
“so-called (…) teams”	Action movie	
“we called (them) COVID-Angels”	Saviors	
“keep swimming, the land is coming”	Enduring an arduous journey	
“be run over”	Action movie	
“fear from perspective of a leader”	Sports team coach	
“we are in the second marathon this year”	Sporting event	
“very very exhausting”	Sporting activity	
“after 3 weeks there is still no coast in sight”	Seafaring	
“declaration of the pandemic”	Public political statement, begin of the battle	Soldier
“to unite everyone behind this flag”	Medieval war scenario	
“And now it’s more like they have twelve different flags. And everyone is hanging out there on their own.”	Medieval war scenario	
“they weaken us, too”	Physical violence	
“under constant fire for months”	War battle	
“my life was over”	Death and dying	Survivor
“absolutely madness”	Anarchy, chaos	
“first six weeks were the hell”	Suffering	
“a monstrous task”	Terrifying, danger	
“every pandemic passes by. That’s how it was in history”	Storytelling, fairy tale	
“this will not be the end of the world now”	Storytelling, fairy tale	
“moving a little closer together again”	Cohesion, safety	
“we have already been through hell and back”	Journey, adventure	
“unlock material costs”	Open a lock	Distributor
“sometimes it is a crazy patchwork”	Processing leftovers	
“to shop (equipment) at the same time, to rebuild it”	Shopping, constructing	
“filling the gaps”	Pouring liquids into gaps	
“I also ordered some additional things that you need”	Shopping	
“the extreme bottlenecks”	Merchandise	
“people cohorted”	Group selection	
“have more people in the pot”	Cooking, preparing	
“selected the physicians who seemed to be the most suitable”	Cooking, preparing	
“every day you squeeze out another one (bed) somewhere”	Pressing out liquid	
“incredible pressure on the system”	Engineering	
“walls are torn down”	Crafting	Craftsman
“to pull up”	Building	
“built many PAX cabinets (IKEA furniture)”	Building	
“being a master at it”	Craft guild	
“we crafted something ourselves”	Building	
“helping clean hand”	Craft guild	
“got to know a new disease”	Learning	Trainee
“during the dark times, in the beginning”	Storytelling, fairy tale	
“getting up and running”	Sports	
“countries that were already badly affected”	Attack	
“brainpower is needed”	Learning, scientific progress	
“tried to look at ways that had gone well”	Learning, experiences	
“the patients were really extremely bad”	Danger, threat	
“rocking along with you”	Companionship	
“that everyone can simply fall back on it”	Danger, accident	
“a journeyman’s piece”	Completion of an apprenticeship	
“it’s all in the drawer”	Packed, secured, hideaway	
“we can activate all this within less than a day”	Operating a system	
“The standard 2 days ago was so old that it (he) has already grown a beard”	Old man, fairy tale	
“I am calm but I had to learn that”	Caring	Parents
“COVID-19-Huddle”	Cheering each other on at a sporting event	
“Let us go and we can do it”	Cohesion	
“they are all doing great,” “unbelievable performance”	Praising children	
“I would not like to put them through that right now either”	Caring	
“in the spring we were the heroes, now we are the bogeymen”	Storytelling, fairy tale	
“And now, Corona, who wants to do it?”	Task attribution to a person	Critic
“the profession is unattractive”	Description of a person	
“we are the fool for everything”	Devaluation	

## 3. Results

Our typology defines characteristics of CM protagonists on four dimensions (Table 2). The dimension “mood” describes the emotional state while the dimension “modus operandi” refers to the specific orientation of the protagonists’ CM actions and interactions with other persons. The third dimension “location” describes the operating space of our protagonists, and the fourth dimension “scope” integrates temporality of perspectives taken by the protagonists (retrospective, present/in-process, or prospective) and enables describing changes through the cyclical structure of the COVID-19 crisis.

**Table 2:** Typology characteristics of CM protagonists.

Type	Mood	Modus operandi	Location	Scope
Adventurer	Enthusiastic	Experiencing events	Journey	Prospective
Trainee	Curious	Learning	School	Prospective
Craftsman	Eager	Crafting, building	Manufactory	Present/in process
Soldier	Resolute	Fighting	Battlefield	Present/in process
Survivor	Resistant	Bearing, escaping	Safety place	Retrospective
Distributor	Rational	Planning, allocating	Headquarter	Prospective
Parent	Prudent	Caring, protecting	Home	Prospective
Critic	Cynical	Reflecting, evaluating	Podium	Retrospective

The protagonists show how clinicians take responsibility for CM in ICUs. They (inter)act in different ways. The order we present the types in this section mirrors their chronological development during the first pandemic year. In the course of data analysis, the majority of the protagonists appeared to us as either male or female. We found that the respondents already gendered the metaphorical repertoire of the protagonists. As a result, six out of eight protagonists are portrayed as gendered.

### 3.1. The adventurer

We found the adventurer protagonist primarily in the baseline interviews. What is remarkable about this protagonist is his enthusiastic mood and motivation in finding CM strategies for the unexpected situation in the ICU, for which he assumes responsibility. By actively searching for an image of the future (“(…) we had no idea what was coming” – C1, 1), he pursues experiencing adventures inspired by action movies (modus operandi). With innovative ideas (“That’s why I invented (…) so-called (…) teams” – C2, 1) and energy to “consistently” combat the pandemic, the adventurer aims to shape his workplace, has effective self-management skills, and restructures processes in teamwork in order to improve them (“we also established something we called COVID-Angels” – C2, 1). He regards CM as a sporting event (“keep swimming, the land is coming” – C2, 2) and he performs the role as a coach (“leader”) who trains his team under time pressure (“breakneck speed” [*Schweinsverlauf*] – C1, 1). He is afraid of being defeated: (“But that was my biggest fear personally as a leader now that we would really be run over” – C2, 1).

The comparison of the baseline and follow-up interviews reveals a change in his prospective orientation (scope) as well as his mood from enthusiastic to pessimistic and exhausted, reflecting increasing routine and persistent workload. “We are now in the second marathon this year. It is very, very exhausting” (C1, 2). We can locate the adventurer in a sporting competition scenario, but he can also be the protagonist in an adventure picture in which he sets out on a journey and swims through a raging river in search of a shore. “How long shall I say, keep swimming, the land is coming. Keep swimming and it does not matter. Do they understand when they are practically breaststroke and paddle for three weeks and it always says the coast is coming soon. And after three weeks there is still no coast in sight” (*sic*) (C2, 2).

### 3.2. The soldier

The soldier is a protagonist who consistently embodies the CM of clinicians during the pandemic. He can be characterized as being brave and resolute (mood). However, the pandemic changes him during his (military) mission on the ICU.

He associates the “declaration of a pandemic” (C3, 1) with the declaration of a war. This figurative analogy symbolizes a life-changing experience in society from the clinicians’ perspective.

For the soldier, CM means going to war to fight for a higher idealistic goal. He experiences his work surrounding as a battlefield scenario in which he orchestrates the staff and guides them through practicing various tactical skills to attack and defend. In preparing for the pandemic, the soldier focuses on building up a team that acts cohesively and effectively “(…) to unite everyone behind this flag, which fortunately also worked out great” (C2, 1). Based on the metaphorical concepts, we perceived a modification of his orientation: in the next phase of the COVID-19 pandemic, he changes his CM strategies from focusing on the team to an individual perspective. This is a strategy for him and his comrades-in-arms to deal with the situation. “And now it’s more like they have twelve different flags. And everyone is hanging out there on their own” (C2, 2).

While at the beginning of the pandemic he feels prepared (among other things by “shutting down regular operations” – C3, 1), in the second phase he experiences less systematic support from decision-makers (“That means they are weakening us, too” – C3, 2). He perceives that he and his comrades are less well equipped and more and more incapable. Possibly this is the reason to focus on himself getting through. The soldier reveals that the work situation (“under constant fire for month” – C2, 2) in particular caused a decreasing team spirit and morale.

The metaphors in the follow-up interviews suggest a persistence of “firing” and show that the soldier and his comrades are permanently in defending action. He has no clear reference to the future; the scope of his actions is embedded in the current situation. He tries to manage it as best as possible. As a fighter, he is defensive and works hard to overcome the war.

### 3.3. The survivor

The survivor is a protagonist who only emerges in the follow-up interviews. He is currently in a safe place. CM is reconstructed negatively, in metaphors of strength and effort, but also marked by an underlying anxiety (“That is, from the ninth of March, my life was over for the time to come.” – C2, 1). The survivor can be characterized as tough and resistant (mood), even though the pandemic took his strength. “I phoned through three batteries from the phone. That was absolute madness [*Wahnsinn*],” “I cannot describe it in any other way, (…) the first six weeks were hell” (C1, 1). We see the survivor as a protagonist who emerges from dealing with impositions. The metaphorical repertoire of this protagonist consists of an iconography of the inconceivable, expressed in images of the monstrous and evil. For him, CM means to overcome “a monstrous task” (C1, 1). The survivor shows a high level of confidence in CM to successfully deal with the crisis together (“moving a little closer together again” – C3, 2). At the same time, he is proud of personal and joint achievements (“After all, we have already been through hell and back.” – C1, 2). His modus operandi is to live on. From this, he often feels powerless and yet prescribes a positive way of thinking, e.g., by repeating affirmations (“Every pandemic passes, so I’m a very positive thinker. Every pandemic passes. That’s how it was in history.” – C1, 2) or by telling a story of comforting endings (“this will not be the end of the world now, (…)” – C3, 2).

### 3.4. The distributor

We found the protagonist of the distributor to be a consistent embodiment of the CM of clinicians during the pandemic and the economic conditions in hospitals. He can be characterized as being engaged and creative, but also rational. He plans and allocates commodities/resources (“unlocks material costs”, “normal patients moved out”- C3,2). He regards CM as a puzzle (“sometimes it is a crazy patchwork [*wahnsinniges Gestückel*]” – C3, 2) and considers his role as “filling the gaps” (C3, 2).

The distributor struggles with having equipment, beds, and human resources available, or not. Since he is prospectively oriented (“scope”), it is important for him to receive “very early and timely this stuff” (C1, 1). “Frankly speaking [*auf Deutsch gesagt*], that saved our asses, and then I also ordered some additional things that you need.” (C1, 1). In the preparation phase, “the extreme bottlenecks [*Engpässe*] in basic deliveries” were very stressful because “the quality decreased significantly as the price increased” (C1, 1), for example in personal protective equipment. “At the moment, we have these filter bags [*Filtertüten*] that you put on there” (C1, 1). The distributor was responsible for the allocation and stocking of the materials – “we had to let people work with it (…)” (C3, 1). Another important point is the acquisition and deployment of (new) employees. He manages the staffing of the units and regulates absence rates. (“(we) have more people in the pot, so to say.” – C3, 2). The distributor uses various possibilities to recruit staff for the ICU, e.g., by offering “experience tours”(C2, 1). He organized patient care (“respond with a lead-in system” – C2, 1) and at the same time “(…) selected the physicians who seemed to be the most suitable (…)” (C1, 1). In the course of the pandemic distribution got more arduous (“In order to cohort the people, a ward block was identified and adapted as a pure corona house” – C1, 1; “every day you squeeze out another one (bed) somewhere” – C3, 2). We have three scenarios for the protagonist “distributor”: first, we can locate him in the headquarters of an industrial company, a location where he can oversee the entire situation. Secondly, he can also sit in front of a puzzle board, or be a cutter in a tailor’s shop. The metaphorical repertoire shows him as a person who tries to be as best as possible in his leadership role with increasing burden at the same time (“incredible pressure on the system” – C3, 2).

### 3.5. The craftsman

The protagonist of the distributor is a close relative of the craftsman. He symbolizes the readiness to contribute to CM with manual skills. By emphasizing building and crafting, he expresses the urgency and the willingness to act especially at the beginning of the COVID-19 pandemic. In this phase of crisis cycle, “walls are torn down” to “pull up” new ICUs (C1, 1). His mood is eager and pleasant and he is located in a manufactory.

The craftsman applies his knowledge of building modular furniture or elements and uses irony to talk about his professional work as a clinician: “I have built many PAX cabinets [*IKEA furniture*] in my life, I’m a master at it, I can do better than pneumology” (C2, 1). He identifies himself with practical activities that are carried out manually (“Finally, we crafted something ourselves” – C1, 1). Hands are presented in their functioning as creative tools of humans. They stand for empowerment to act. The image of a helping hand was adapted to a “helping clean hand” (C2, 1). It characterizes the craftsman’s hygiene-compliant work in the hospital setting and represents a high-quality standard and a respectful treatment of patients. This protagonist obeys the traditions and virtues of his guild. He aims to manufacture a product that is useful in everyday life (e.g., furniture or architectural components). His modus operandi pursues the construction and reconstruction of high-functional elements. From this perspective, manual contribution is temporally limited (scope), as it is intended only to overcome a crisis in an unexpected scenario. Craftsmanship is not oriented towards technical progress, but it is used to process the resources that have been distributed in a high-quality way. In doing so, the craftsman is highly motivated and engaged.

### 3.6. The trainee

We found the protagonist of the trainee only in the baseline interviews. She is curious with a prospective modus operandi of learning (“We got to know a new disease” – C1, 1). She embodies acquiring knowledge under intense time pressure by gaining enlightenment “during the dark times, in the beginning, when none of us knew what COVID-19 was” (C2, 1). The protagonist “trainee” symbolizes the willingness to contribute to CM with new expertise and to learn during and from the pandemic. Metaphors of activity like “getting up and running” (C1, 1) express the fast pace of change and readiness to act, especially at the beginning of the pandemic.

In this crisis phase, “brainpower [*Gehirnschmalz*] is needed,” and “teaching and training” to form strategies and new routines. That means she tried “to get as much information as possible from the countries that were already badly affected” (C2, 1) by watching many interviews from Bergamo in northern Italy. She “tried to look at ways that had gone well (C2, 1)” and screened research papers. Her mood is motivated, yet guided by concern, “especially since the patients were really extremely bad. And that is a point that we had to learn” (C1, 1).

The image of “rocking along with you” (C1, 1) characterizes the trainee (in leadership role). She wants to support and empower their employees – “that everyone can simply fall back on it and also know what is meant by it, also understand it” (C2, 1). Consequently, she associates learning with intellectual aspirations. The metaphors in the follow-up interviews describe CM as “a journeyman’s piece [*ein Lehrstück*]” (C3, 2) as is required on completion of a professional education. It suggests that new routines are developed and professionalized (“It’s all in the drawer [*Schublade*]”- C2, 2). That is perhaps also a difference. “We are much better prepared and have also learned that we can activate all this within less than a day” (C2, 2). From our point of view, the trainee is a lively and curious person who is in a school scenario. In this environment, she is confronted with information that quickly becomes outdated. “The standard two days ago was so old that it (he) has already grown a beard” (C1, 1). The metaphorical concept provides associations with a fairy tale scenario. Thus, at the end of a fairy tale, the reader is always entrusted with the message of a morale. In the crisis story of the pandemic, the trainee learned the lesson of effective preparation to take responsibility for her staff.

### 3.7. The parents

The parents are protagonists we found both in the baseline and in the follow-up interviews in a prudent mood. For parents, CM means caring and protecting each other; employees become their children (“already worried” – C3, 1/2). Parents evaluate the current conditions, and care on outcomes in the future (prospective scope) (“I do not sleep well” – C3, 2).

However, the pandemic changed the role of mothers/fathers. At the beginning, we observe parents who are concerned about health protection: “So now that I know, well, these are the best-protected employees, I am calm, but I had to learn that” (C1, 1). They reveal that it was important to build a sense of belonging to a community (“COVID-19-Huddle,” “Let us go and we can do it” – C2, 1).

In the follow-up interviews, they are concerned about the workload. The employees “(…) (have to) do things now that we would not usually make them (to) do. And I would not like to put them through that right now either, but there’s no other way and they are all doing great” (C3, 2). Parents feel responsible and helpless at the same time. They “put them (employees) in work situations where (the parents) know that they cannot do their job in the best possible way. (…) firstly, we overwork the employees, secondly, they go home and have the feeling ‘I could not do my job optimally today’. Yes, and thirdly, they are always afraid of being infected” (C3, 2). Parents are proud of their children (employees) (“unbelievable performance” – C3, 2) and emphasize “to strengthen the team” (C3, 2). At the same time, they feel devalued by their fellow citizens, and perceive these reactions with skepticism and concern. “(…) in the spring we were the heroes, now we are the bogeymen for everything. So we are the bogeyman for everything, really” (C1, 2). We perceive parents in a conflicted life phase scenario. On the one hand, they intend to give their children the freedom to unfold freely, but on the other hand, they worry about them because of the evils of the world. In addition, they are disappointed that they receive little recognition for their achievements (care work) in society.

### 3.8. The critic

In the follow-up interviews, we found metaphors that emphasized the protagonist “critic” more explicitly. This person reflects on operating costs and hospital financing in the German health system. The critic is unisex. We used the singular they.

The critic introduces a meta-perspective on the system (“we are still relatively fine in Germany compared to other countries” – C3, 2) and on themselves as part of it (“Yes, welcome to this world” – C2, 2). They monitor and evaluate new policies regarding the COVID-19 crisis with attention to the ongoing crisis in hospitals. The mood is “bitter” and cynical, the perspective is rather pessimistic and “worried” (C2, 2). They understand the pandemic as a huge, hard-to-solve task that coincides with an ongoing crisis in health systems (“And now, Corona, who wants to do it?” – C1, 2).

The viewpoint is detached from daily routines and enables taking up a transcendent position. They use critical voice as an evaluative tool to reflect on coinciding crises and thus compensate frustration. That is why another possible scenario of the critic is working as a staff council. As an advocate for patients and ICU staff, they shed light on the economic strategy of the healthcare system, while the work within is characterized as being physically challenging and brutal (“if we operate on a hip, we get much more money” – C2, 2). Besides a lack in equipment, they also critically mention the decreasing human resources in hospitals. They consider connections between workload and structural working conditions and the attractiveness of health professions (“The profession is unattractive. Weekends, nights, heavy workload” – C1, 2). In the leadership role, they oscillate between employees and hospital management. This leads to contradictory and ambivalent situations, because they refer to both positions. Therefore, “job” is personified as a not-appropriate romantic partner with whom a person spends a lot of time within their life. Similar to a dysfunctional relationship, the critic remains in the ambivalent role. He mediates between hospital management and staff.

The protagonist assesses current crisis conditions in hospitals, but does not have suitable suggestions for transformation, which is why their scope is limited. To make a comparison with the continuing situation (“we are the fool for everything” – C1, 2), they refer to events that lie in the past (“I mean, honestly, in 2008, the banks were propped up, too” – C3, 2). The scope is primarily retrospective or destructive, when imagining the future (“I do not think we are well prepared” – C3, 2). Their actions (reflecting, evaluating, and criticizing) depend on the system which is criticized. In case of a system change, the critic’s mission will be fulfilled and this protagonist would disappear, at least until the next critic-worthy situation occurs.

## 4. Discussion

In our study we used metaphor analysis to explore images of CM within the metaphorical speech of ICU clinicians in leadership roles. We illustrated how metaphorical language helped clinicians in leadership to make sense of their experiences in coinciding and recurring crises. To contrast differences in the use of metaphors, we selected three interviewees from our original study (38). The metaphor analysis yielded a typology of eight self-images (protagonists) for the CM of ICU clinicians in leadership: the adventurer, the soldier, the survivor, the distributor, the craftsman, the trainee, the parents, and the critic. The protagonists are reconstructed personifications and embody different qualities of a leader; they refer to intra- and inter-role conflicts within multiple crisis conditions. The craftsman, for example, shows a constructive way of dealing with the current situation of coinciding crises – working with limited resources while maintaining day-to-day business. The way in which the protagonists emerge and disappear over time reveals changes in perspectives, orientations, actions, and emotions of the clinicians we interviewed. Some of the protagonists disappear after a pandemic crisis cycle at the beginning, while others are introduced as the pandemic routine of clinical practice is established. For example, an initially enthusiastic adventurer turns into a rather disillusioned survivor and, with increasing expertise, a trainee becomes a soldier or even a survivor over time. Thus, some of the protagonists are dependent on or related to each other. It is only with the experiences of the hardworking craftsman, the rational perspective of the distributor, or the viewpoint of the caring mother/father that the critic can undertake a reflection on the coinciding crises they experienced. The same applies, for example, to the distributor and the craftsman. The distributor plans and allocates resources, while the craftsman applies them to manufacture a product. Protagonists’ internal changes do not necessarily lead to a new type as long as basic characteristics (modus operandi and scope) remain constant. However, mood and perception may change over time. For example, the more experiences the parent protagonists have on the ward, the more they are concerned about the children (staff). The modus operandi of care and support remains the same. As further examples, the adventurer shows less enthusiasm in the follow-up interview and the soldier gives up his team orientation in fighting and focuses more on himself. It is unclear whether our protagonists of CM will (re)appear in the future, especially considering the uncertain development of the crises. Possibly structures will be reorganized in the coming months and protagonists like the craftsman will appear again. Even the distributor will regain importance in case of a new pandemic crisis cycle. In contrast, we consider it unlikely that the adventurer and the soldier will reappear in this cycle. It is possible that they have run out of steam over the course of time.

We applied the metaphor analysis separately to the original study (38), and did not perform a method triangulation, but instead generated a new study with independent objectives. Empirically, we succeeded in exploring how the metaphorical language in three cases changed during the first year of the pandemic. The metaphorical repertoire in the data is likely influenced and contaminated with metaphors that were already established before the pandemic. The use of military metaphors has been observed in previous health crises, e.g., during influenza epidemics and the AIDS crisis (41, 46). Since the beginning of the COVID-19 pandemic, we have witnessed its recurrent use by political representatives and the media, especially on television (47). Military metaphors are also frequently used by both medical staff and patients. Thus, it is not surprising that we found the soldier as a protagonist of clinicians’ crisis management (37, 48, 49). During the analysis, we had to consider the societal context. Throughout the pandemic, changes in the origin of metaphors (source areas) occurred, i.e., metaphorical speak of public discourses was/is as dynamic as the pandemic itself (50). However, our own viewpoints and associations changed as well, which impacted data interpretation. Local metaphorical concepts may have different meanings across individuals, groups, and cultures (51). Becoming aware of this was a fundamental condition for being able to identify metaphors in the data. It was also a challenge for us to translate metaphors adequately into English in their culturally specific semantic function. Our strategy for this article was to find equivalents in English and to show original expressions in square brackets. It would have been interesting to examine further heterogeneous examples beyond the German context.

With two exceptions, the protagonists are gendered. They embody professions or occupations that are traditionally ascribed as male or female in Western societies (craftsman, soldier, educator, and caregiver) (52, 53). Although we did not intend to underpin a binary gender perspective, we noticed that the metaphorical repertoire of these protagonists was already gendered by the interviewees. That is why the protagonists appeared masculine and feminine to us throughout the data analysis. Historically, health professions were gendered, and beliefs about gender came to be embedded in professional work. Thus, gender is still relevant in many ways. Studies document numerous challenges to women pursuing careers in male-dominated fields (54). We point out that attributing metaphorical concepts to masculine and feminine involves the risk of reproducing a gender binary bias, instead of understanding gender as socially constructed (41). We also portrayed two protagonists in the generic third person “they.” Parents appear as a dyad of mother and father, while the protagonist critic appears as gender-fluid.

Our study was conducted in the context of the German healthcare system. Compared to other countries, there is a perceived need for reforms in terms of hospital structure and financing and, above all, an increasing shortage of nurses (55). Our findings provide insights into the pandemic working conditions of ICU clinicians in leadership positions, especially into their responsibilities and burdens (56, 57). However, they also reflect concepts and traits that are socio-culturally constructed, whether in relation to the German culture or to the medical setting in general. The trainee reflects a healthcare system in which training evidenced by certificates, diplomas, exams, and titles is seen as essential for ensuring quality of care. The distributor embodies a type of socialized medicine (related to the principle of solidarity (58), one of the key characteristics of German healthcare system). In particular, the protagonists “parents” and “critic” show their disappointment about the lack of social recognition of their achievements and the poor structural working conditions. The critic reflects a system under constant pressure, in which challenges, such as economic pressure (hospital financing), lack of digitalization, and resource shortages, put clinicians and hospital managers in conflict with current care needs and ethical duties (55, 59, 60). Literature on the burden of coping with the pandemic shows differences between professions (physicians and nurses). Durgun et al. (61) reflect on nurses’ perceptions of the COVID-19 pandemic. The metaphors mentioned by nurses included, e.g., living organisms, emotion, danger, and death. Most expressions indicate hopelessness (61). Naamati-Schneider and Gabay (37) analyzed metaphors and their role in framing reality and shaping coping mechanisms among COVID-19 ward directors during the first wave of the pandemic in Israel. Effective coping was facilitated by war metaphors that communicated a sense of purpose and meaning at both the organizational and individual level (37). In contrast to our study, these two studies reveal only momentary observations. Our metaphor analysis shows transitions in experiencing crises in the first year of the pandemic by metaphorical speech. The typology embodies implicit emotions, norms, values, and attitudes related to CM and depicts changes in the mental state of ICU clinicians over a longer time. At the beginning of the pandemic, ICU clinicians had to be able to switch between different roles without the possibility to prepare for it. The exceptional situation increased the importance of leadership and resulted in new and parallel tasks, e.g., updating the state of knowledge on COVID-19 and taking into account policy regulations, in addition to being involved with patient care (62, 63). Moreover, it was impossible for senior clinicians to object to these tasks. Studies showed that inadequate working conditions, such as lack of supplies, equipment, or staff, lead to a decrease in motivation (64). However, the participants in our study were highly motivated for CM, especially at the beginning of the pandemic. We assume that the exceptional situation of the pandemic strengthened the cohesion in ICU teams. They developed the motivation to manage the situation together.

As the use of metaphors of CM changed during the first year of the pandemic, we encourage further research on how the pandemic and staff shortages, not only in ICUs, affect leadership self-image in the long-term. We showed that concepts and perceptions of CM might strongly differ between and within leaders as well as over time. This has implications for policy and practice, particularly for human resource management of hospitals and pandemic preparedness. In order to support clinicians in their role as leaders, it could be very important to consider their self-image and to acknowledge the differences and changes in self-images of leadership personnel in crisis situations as in general. This highlights the urgent need for policy and practice decision-makers to better understand the challenges associated with leadership in crisis and preparedness and to support clinicians in performing their leadership roles.

## Data availability statement

The raw data supporting the conclusions of this article will be made available upon request by the authors, without undue reservation.

## Ethics statement

The studies involving human participants were reviewed and approved by Ethics committee at Otto von Guericke University Magdeburg, Faculty of Medicine (51/20) as well as University of Regensburg (20-1771-101). The participants provided their written informed consent to participate in this study.

## Author contributions

JP: formal analysis, investigation, methodology, validation, visualization, writing/original draft, and writing/review and editing. MH: formal analysis, investigation, methodology, validation, visualization, writing/original draft, and writing/review and editing. SB: writing/review and editing. K-PD: project administration, data curation and writing/review and editing. IH: investigation and writing/review and editing. RS: methodology and writing/review and editing. CA: conceptualisation, funding acquisition, project administration, supervision, and writing/review and editing. All authors contributed to the article and approved the submitted version.

## Funding

The authors disclose receipt of the following financial support for the research, authorship, and/or publication of this article: this work was supported by intramural funding.

## Conflict of interest

The authors declare that the research was conducted in the absence of any commercial or financial relationships that could be construed as a potential conflict of interest.

## Publisher’s note

All claims expressed in this article are solely those of the authors and do not necessarily represent those of their affiliated organizations, or those of the publisher, the editors and the reviewers. Any product that may be evaluated in this article, or claim that may be made by its manufacturer, is not guaranteed or endorsed by the publisher.
